# Modeling Signal-to-Noise Ratio of CMOS Image Sensors with a Stochastic Approach under Non-Stationary Conditions

**DOI:** 10.3390/s23177344

**Published:** 2023-08-23

**Authors:** Gil Cherniak, Jonathan Nemirovsky, Amikam Nemirovsky, Yael Nemirovsky

**Affiliations:** 1 Electrical Engineering Department, Technion—Israel Institute of Technology, Haifa 3200003, Israel; gilcher@campus.technion.ac.il (G.C.); jnemirov@technion.ac.il (J.N.); 2 Department of Electrical Engineering, Kinneret College on the Sea of Galilee, Tzemah 1513200, Israel; amikam.nemirovsky@mx.kinneret.ac.il

**Keywords:** CMOS image sensor, gated imaging, signal-to-noise ratio (SNR)

## Abstract

A stochastic model for characterizing the conversion gain of Active Pixel Complementary metal–oxide–semiconductor (CMOS) image sensors (APS), assuming stationary conditions was recently presented in this journal. In this study, we extend the stochastic approach to non-stationary conditions. Non-stationary conditions occur in gated imaging applications. This new stochastic model, which is based on fundamental physical considerations, enlightens us with new insights into gated CMOS imaging, regardless of the sensor. The Signal-to-Noise Ratio (SNR) is simulated, allowing optimized performance. The conversion gain should be determined under stationary conditions.

## 1. Introduction

Gated imaging is an active imaging technique using a laser light source for seeing objects at desired distances. Gated imaging is suitable for various applications such as defense, security, automotive, and robots for consumer electronics [1,2,3,4,5].

Time-gated measurement is a class of time-of-flight ranging or imaging technologies where a sensor with tightly controlled opening and closing times of the shutter is used in coincidence with a high-power pulsed light source. Signal-to-noise ratio (SNR) is enhanced by time-gated technique since it limits the exposure time of the sensor to the return time of an emitted light pulse from an object at a defined distance r [6,7,8,9,10].

The time-gated technologies may be divided into two distinct classes: single-shot and multi-shot. For example, single-shot light detection and ranging (Lidar) captures the returned light from one single light pulse while multi-shot LIDAR integrates the returned light pulses during several laser periods. Furthermore, the implementation of the time-gated sensing may be based on two different gating modes: software gating and hardware gating.

In this work, we address active gating where the sensor is a CMOS Image sensor, as described in our previous paper [11]. Gating is used for depth measurements and to achieve 3D imaging. Non-stationary conditions occur in such applications. Therefore, the stochastic approach presented in ref [11] needs to be re-defined.

Section 2 presents the basic operation model of the gating approach. Section 3 presents the non-stationary stochastic modeling. The simulation of the signal-to-noise ratio (SNR) evaluated with the non-stationary stochastic approach is presented in Section 4. Section 5 summarizes the results.

## 2. The Gated Imaging Basic Operation Model

**Figure 1:**
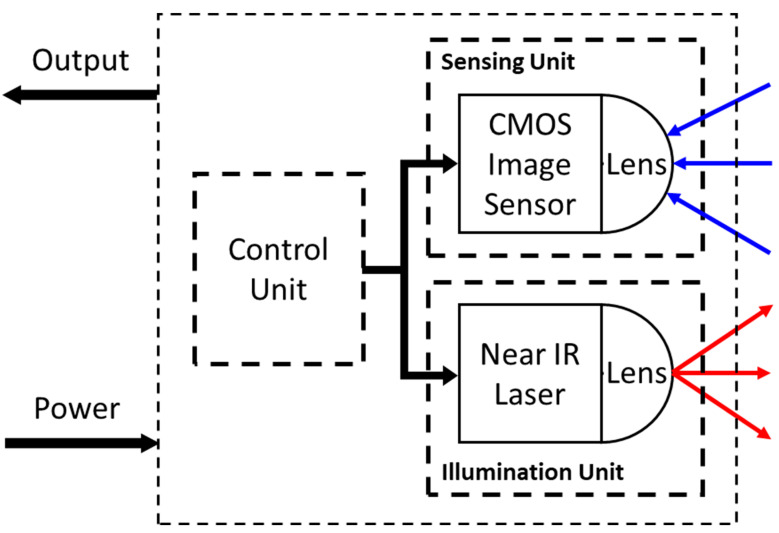
A schematic block diagram of the Gated Imaging system. The system consists of two main modules—the illumination unit, built of a Near Infra-Red (IR) laser, which emits the laser pulse (marked in red), and the CMOS Image sensor, which receives the reflected light (marked in blue). A control unit controls both units, gating each unit according to the timing diagram.

The basic principle of gated imaging was described earlier for various applications [12,13]. A camera and a pulsed laser are placed close to each other. The idea is to send a pulse of light and hold the camera in the off position until an optical echo from a specific range (r) returns to the camera, as shown in Figure 1.

The advantages of gated imaging are obvious. Photons, traveling at the speed of light, that are reflected at different ranges, arrive at different points in time. The imaging sensor or camera only “opens the gate” (i.e., starts the exposure) after a certain time delay for a very short period. Therefore, the sensor is not affected by scattered photons or parasitic light sources. Only the photons that arrive within the right period contribute to the resulting image. The time delay (t) determines the depth measurement range according to r = ct/2, where c is the speed of light. Therefore, the resulting image consists of information only from reflected photons at the distance of interest.

Active gated-imaging technology incorporates range-gating technology, based on multiple “Time-of-Flight” events per single read-out image frames in the sensor. Active gating technology combines two key components: a pulsed light source (i.e., Vertical Cavity Surface Emitting Laser (VCSEL)) and a specially designed gated sensor that exposes and blocks light at high speeds of the order of nanoseconds up to microseconds. Each reflected light source pulse is accumulated in the gated sensor based on a specific “Time-of-Flight” event. Once a desired signal (corresponding to a desired depth-of-field) is accumulated in the sensor, the image is read out. This method provides high SNR with relatively low peak power illumination.

A system employing such a gated timing is shown in Figure 1.

**Figure 2:**
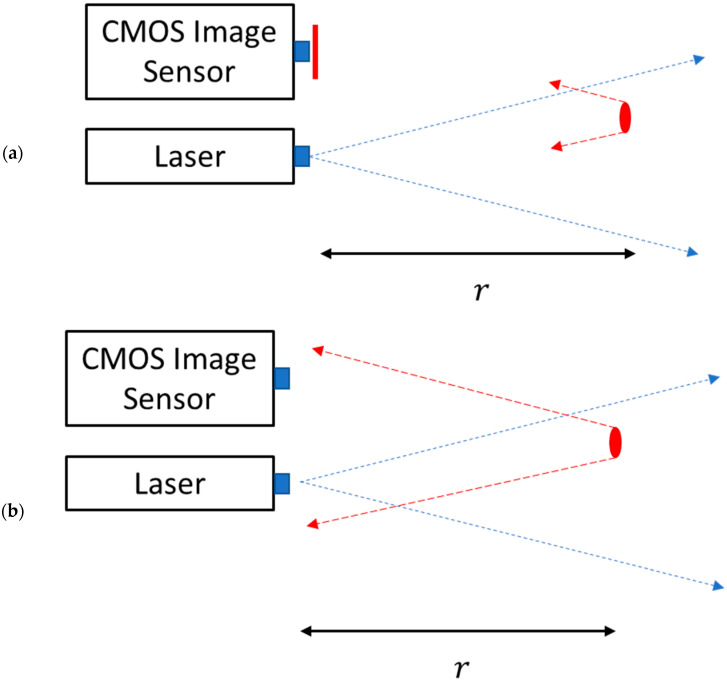
A pulse, marked in a blue dashed line, is emitted towards the red object, at a distance r. Once the pulse reaches the object, it is reflected toward the sensor. (**a**) The shutter on the CMOS Image sensor is closed, the shutter (appears in red) is covering the sensor so no light can enter. (**b**) After a certain time, measured from the pulse sending, it arrives at the sensor and the shutter is opened. The shutter stays open for a period. The laser pulse duration is not shown in the figure.

Figure 2 demonstrates pulse propagation from and towards the sensor. For the sake of clarity, we assume that the laser pulse as well as the gating time are rectangular. Real laser pulses are Gaussian, and the edges could be described by the error function. This would just make the expressions in Section 3 more complicated, without getting new physical insight.

## 3. Mean and Variance of the Output Voltage in Non-Stationary Regimes

### 3.1. Mathematical Setup

The relations between the mean and variance of the random process representing the output voltage obtained for an arbitrary time-varying illumination are discussed here.

**Figure 3:**
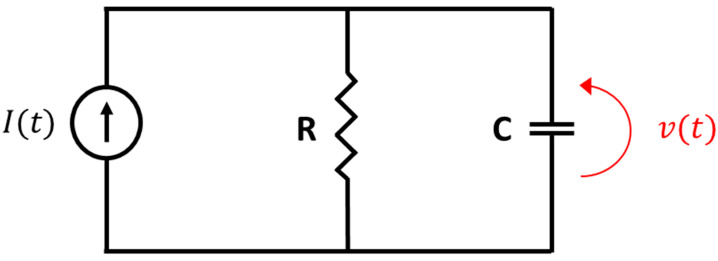
The electrical network model of the sensing junction. A current source *I(t)* injects current to the circuit. C represents the junction capacitance and R represents the effective resistance of the junction. The voltage over the capacitor C is *v(t)*, which appears in red.

The equivalent electrical circuit of the photodiode assumed here is described in Figure 3 and composes a random current source I(t) that injects electrons into the output impedance of the photodiode, namely, a capacitor C in parallel with a resistor R, both assumed to be constant.

For the non-stationary case, the random current injected by the photodiode is described as:

(1) $$I(t)=q_{0}\sum_{k=-\infty }^{\infty }X_{k}\cdot \delta (t-t_{k})$$

where $q_{0}$ is the electronic charge, δ(⋅) the Dirac function, $\left\{t_{k}\right\}$ the center points of narrow and non-overlapping time intervals denoted by $\left\{\Delta t_{k}\right\}$, and that overall covers the entire time under inspection. The random reality is here introduced by a series of mutually independent random variables $\left\{X_{k}\right\}$, where each random variable $X_{k}$ is equal to the number of injected electrons within the corresponding time interval $\Delta t_{k}$. When $\Delta t_{k}\to 0$ $X_{k}$ one may assume only the values 1 or 0, where the probability for the value 1 is determined by an underlying time-function (commonly known as the Illumination function) ϕ(t), such that for $\Delta t_{k}\to 0$, $P\left\{X_{k}=1\right\}=\phi (t_{k})\cdot \Delta t_{k}$ while $P\left\{X_{k}=0\right\}=1-\phi (t_{k})\cdot \Delta t_{k}$.

Hence $E\left\{X_{k}\right\}=E\left\{X_{k}^{2}\right\}=\phi (t_{k})\cdot \Delta t_{k}$, so that ϕ(t) represents the expected number of electrons injected by the photodiode per unit time, owing to some external light source/sources. Since the number of injected electrons within non-overlapping time intervals are independent, it is apparent that when $\Delta t_{k}\to 0$ and $\Delta t_{j}\to 0$,

(2) $$E\left\{\left(X_{k}-\phi (t_{k})\Delta t_{k}\right)\cdot \left(X_{j}-\phi (t_{j})\Delta t_{j}\right)\right\}\to \left\{\begin{array}{c} \begin{array}{cc} E\left\{X_{k}^{2}\right\}=\phi (t_{k})\Delta t_{k} & if\;j=k \end{array}\\ \begin{array}{cc} 0 & if\;j\neq k \end{array} \end{array}\right\}$$

The temporal voltage across the photodiode can be represented as the response of the current charging the junction capacitance, using a typical capacitor charging response function, being charged at a random time $T_{k}$ with $\alpha ={\left(RC\right)}^{-1}\left[s^{-1}\right]$ and u(t) the unit step function:

(3) $$h\left(t\right)=\frac{q_{0}}{C}\cdot u\left(t\right)\cdot \exp(-\alpha \cdot t)$$

It follows from Equations (1) and (3) that the temporal voltage across the photodiode output can be represented as a random process:

(4) $$V(t)=\frac{1}{C}\int_{-\infty }^{t}I\left(\vartheta \right)*h(\vartheta )\cdot d\vartheta =\frac{q_{0}}{C}\sum_{k=-\infty }^{\infty }X_{k}\cdot u(t-t_{k})\cdot \exp\{-\alpha \cdot (t-t_{k})\}$$

The accumulating time-integral of this voltage S(t) is the random process:

(5) $$S(t)=\int_{-\infty }^{t}V(\vartheta )\cdot d\vartheta =q_{0}R\cdot \sum_{k=-\infty }^{\infty }X_{k}\cdot u(t-t_{k})\cdot \left[1-\exp\{-\alpha (t-t_{k})\}\right]$$

Equations (4) and (5) describe two different cases of voltage measurement. Equation (4) describes the temporal voltage measurement (case 1) while Equation (5) describes the integrated voltage measurement (case 2).

### 3.2. The Mean and Variance of V(t) and S(t)

The mean and variance of both V(t) and S(t) are important for analyzing the performance of the measurement. Since $E\{X_{k}\}=\phi (t_{k})\cdot \Delta t_{k}$, using Equation (4), the mean of V(t) is:

(6) $$\begin{array}{c} m_{V}(t)=E\{V(t)\}=\frac{q_{0}}{C}\sum_{k=-\infty }^{\infty }\phi (t_{k})\cdot \Delta t_{k}\cdot u(t-t_{k})\cdot \exp\{-\alpha (t-t_{k})\}\overset{\;\Delta t_{k}\to 0\;}{\to }\\ \frac{q_{0}}{C}\int_{-\infty }^{\infty }\phi (\vartheta )\cdot u(t-\vartheta )\cdot \exp\{-\alpha (t-\vartheta )\}\cdot d\vartheta =\\ =\frac{q_{0}}{C}\cdot \exp\{-\alpha t\}\cdot \int_{-\infty }^{t}\phi (\vartheta )\cdot \exp\{\alpha \vartheta )\}\cdot d\vartheta \end{array}$$

Likewise, using Equation (5), the mean of S(t) is:

(7) $$m_{S}(t)=E\left\{S(t)\right\}=q_{0}R\cdot \sum_{k=-\infty }^{\infty }\phi (t_{k})\cdot \Delta t_{k}\cdot u(t-t_{k})\cdot \left[1-\exp\{-\alpha (t-t_{k})\}\right]$$

Which, upon letting $\Delta t_{k}\to 0$, becomes:

(8) $$\begin{array}{c} m_{S}(t)=E\left\{S(t)\right\}=q_{0}R\cdot \int_{-\infty }^{\infty }\phi (\vartheta )\cdot u(t-\vartheta )\cdot \left[1-\exp\{-\alpha (t-\vartheta )\}\right]\cdot d\vartheta =\\ =q_{0}R\cdot \int_{-\infty }^{t}\phi (\vartheta )\cdot \left[1-\exp\{-\alpha (t-\vartheta )\}\right]\cdot d\vartheta =\\ =q_{0}R\cdot \exp\{-\alpha t\}\cdot \int_{-\infty }^{t}\phi (\vartheta )\cdot \left[\exp\{\alpha t\}-\exp\{\alpha \vartheta \}\right]\cdot d\vartheta \end{array}$$

Next, using Equation (2), the variance of V(t) can be expressed as:

(9) $$\sigma_{V}^{2}(t)=E\left\{{\left[V(t)-m_{V}(t)\right]}^{2}\right\}=\;=E\left\{{\left[\frac{q_{0}}{C}\sum_{k=-\infty }^{\infty }\left(X_{k}-\phi (t_{k})\cdot \Delta t_{k}\right)\cdot u(t-t_{k})\cdot \exp\{-\alpha (t-t_{k})\}\right]}^{2}\right\}=\;={\left(\frac{q_{0}}{C}\right)}^{2}\cdot \sum_{j,k=-\infty }^{\infty }E\left\{\left(X_{j}-\phi (t_{j})\cdot \Delta t_{j}\right)\cdot \left(X_{k}-\phi (t_{k})\cdot \Delta t_{k}\right)\right\}\cdot u(t-t_{j})\cdot u(t-t_{k})\cdot \exp\{-\alpha (2t-t_{j}-t_{k})\}=\;={\left(\frac{q_{0}}{C}\right)}^{2}\cdot \sum_{k=-\infty }^{\infty }\phi (t_{k})\cdot \Delta t_{k}\cdot u(t-t_{k})\cdot \exp\{-2\alpha (t-t_{k})\}\overset{\;\Delta t_{k}\to o\;}{\to }\;{\left(\frac{q_{0}}{C}\right)}^{2}\cdot \int_{-\infty }^{\infty }\phi (\vartheta )\cdot u(t-\vartheta )\cdot \exp\{-2\alpha (t-\vartheta )\}\cdot d\vartheta =\;={\left(\frac{q_{0}}{C}\right)}^{2}\cdot \exp\{-2\alpha t\}\int_{-\infty }^{t}\phi (\vartheta )\cdot \exp\{2\alpha \vartheta \}\cdot d\vartheta$$

Likewise, from Equations (2), (5) and (7) the variance of S(t) is:

(10) $$\begin{array}{c} \sigma_{S}^{2}\left(t\right)=E\left\{{\left[S\left(t\right)-m_{S}\left(t\right)\right]}^{2}\right\}=\\ ={\left(\frac{q_{0}}{C}\right)}^{2}\cdot \sum_{j,k=-\infty }^{\infty }E\left\{\left(X_{j}-\phi \left(t_{j}\right)\right)\cdot \left(X_{k}-\phi \left(t_{k}\right)\right)\right\}\cdot u\left(t-t_{j}\right)\cdot u\left(t-t_{k}\right)\cdot \left[1-e^{-\alpha \left(t-t_{j}\right)}\right]\cdot \left[1-e^{-\alpha \left(t-t_{k}\right)}\right]=\\ ={\left(\frac{q_{0}}{C}\right)}^{2}\cdot \sum_{k=-\infty }^{\infty }\phi \left(t_{k}\right)\cdot \Delta t_{k}\cdot u\left(t-t_{k}\right)\cdot [1-e^{-\alpha \left(t-t_{k}\right)}{]}^{2}\overset{\;\Delta t_{k}\to 0\;}{\to }\\ {\left(\frac{q_{0}}{C}\right)}^{2}\cdot \int_{-\infty }^{\infty }\phi \left(\vartheta \right)\cdot u(t-\vartheta )\cdot [1-e^{-\alpha \left(t-\vartheta \right)}{]}^{2}\cdot d\vartheta \\ ={\left(\frac{q_{0}}{C}\right)}^{2}\cdot \int_{-\infty }^{t}\phi \left(\vartheta \right)\cdot [1-e^{-\alpha \left(t-\vartheta \right)}{]}^{2}\cdot d\vartheta \end{array}$$

### 3.3. Signal-to-Noise Ratio of V(t) and S(t)

The ratio ${\left(\frac{S}{N}\right)}_{V}=\frac{m_{V}(t)}{\sigma_{V}(t)}$ and ${\left(\frac{S}{N}\right)}_{S}=\frac{m_{S}(t)}{\sigma_{S}(t)}$ are dimensionless quantities that have special significance to the qualities of the measurement. From the expressions presented above it follows that for case 1:

(11) $${\left(\frac{S}{N}\right)}_{V}=\frac{m_{V}(t)}{\sigma_{V}(t)}=\frac{\int_{-\infty }^{t}\phi (\vartheta )\cdot \exp\left\{\alpha \vartheta \right\}\cdot d\vartheta }{{\left[\int_{-\infty }^{t}\phi \left(\vartheta \right)\cdot \exp\left\{2\alpha \vartheta \right\}\cdot d\vartheta \right]}^{1/2}\;}$$

Hence, in the special case where the illumination function is a step function $\phi (t)=\phi_{0}\cdot u(t)$,

(12) $${\left(\frac{S}{N}\right)}_{V}=\frac{m_{V}(t)}{\sigma_{V}(t)}=\frac{\int_{0}^{t}\phi_{0}\cdot \exp\left\{\alpha \vartheta \right\}\cdot d\vartheta }{{\left[\int_{0}^{t}\phi_{0}\cdot \exp\left\{2\alpha \vartheta \right\}\cdot d\vartheta \right]}^{1/2}}={\left[\frac{2\phi_{0}}{\alpha }\cdot \frac{\exp(\alpha t)-1}{\exp(\alpha t)+1}\right]}^{1/2}\;={\left[\frac{2\phi_{0}}{\alpha }\cdot \tanh\left(\frac{\alpha t}{2}\right)\right]}^{1/2}$$

In principle, the signal-to-noise ratio increases with the duration of the measurement but approaches the limiting value ${\left(2\phi_{0}\cdot \alpha^{-1}\right)}^{1/2}$ when 1≪αt.

When the measured quantity is S(t) (case 2) the signal-to-noise ratio is:

(13) $${\left(\frac{S}{N}\right)}_{S}=\frac{m_{S}(t)}{\sigma_{S}(t)}=\frac{\int_{-\infty }^{t}\phi (\vartheta )\cdot \left[1-e^{-\alpha (t-\vartheta )}\right]\cdot d\vartheta }{{\left[\int_{-\infty }^{t}\phi \left(\vartheta \right)\cdot {\left[1-e^{-\alpha \left(t-\vartheta \right)}\right]}^{2}\cdot d\vartheta \right]}^{1/2}}$$

Which, for the above step function illumination is:

(14) $${\left(\frac{S}{N}\right)}_{S}=\frac{m_{S}(t)}{\sigma_{S}(t)}=\frac{\int_{0}^{t}\phi_{0}\cdot \left[1-e^{-\alpha (t-\vartheta )}\right]\cdot d\vartheta }{{\left[\int_{0}^{t}\phi_{0}\cdot {\left[1-e^{-\alpha \left(t-\vartheta \right)}\right]}^{2}\cdot d\vartheta \right]}^{1/2}}=\;=\sqrt{\phi_{0}}\cdot \frac{\int_{0}^{t}\left[1-e^{-\alpha (t-\vartheta )}\right]\cdot d\vartheta }{{\left[\int_{0}^{t}{\left[1-e^{-\alpha \left(t-\vartheta \right)}\right]}^{2}\cdot d\vartheta \right]}^{1/2}}=\sqrt{\phi_{0}}\cdot \sqrt{\frac{2\cdot {\left[t-\alpha^{-1}(1-e^{-\alpha t})\right]}^{2}}{\left[2t-4\alpha^{-1}(1-e^{-\alpha t})+\alpha^{-1}(1-e^{-2\alpha t})\right]}}$$

In this case the signal-to-noise ratio increases with the measurement duration, however, in this case without limit and for 1≪αt it becomes asymptotically equal to ${\left(\phi_{0}\cdot t\right)}^{1/2}$.

## 4. Numerical Modeling and Simulation

The equivalent electrical network in Figure 3 represents the capacitor C in which photo-carriers are being stored, while its parallel resistor R represents the equivalent resistance. An illumination pulse is transmitted towards an object, reflected from it, and then captured by the camera’s photodiode, where it is transformed into photo-carriers and stored in capacitor C. The illumination pulse shape presented in our model is the pulse reflected from the object. Measuring the voltage can be completed in two main approaches, described earlier as case 1, where temporal voltage is being measured, and case 2, where an integrated voltage is being measured.

The model divides the pulse into small time intervals $\Delta t_{k}$, such that in every time interval there is a probability for creation of a single photo-carrier, or none. The probability for the creation of more than a single photo-carrier in time interval $\Delta t_{k}\to 0$ is negligible. The model is time-dependent and represents the SNR value at the time within the integration time. Time *t* = 0 represents the shutter opening. It is assumed that the time t is smaller than the exposure time of the frame.

The first method (case 1) considers the temporal voltage on the capacitor during the measuring time. This method applies when the illumination pulse is very narrow, so that in a short period of time, many photo-carriers are collected in the photodiode. This method can be used in SPADs (Single Photon Avalanche Diode) and Silicon multipliers, where the firing is detected “instantly” [6].

The second method (case 2) is accumulative, which integrates the voltage on the capacitor during the integration time. This method applies for wider pulses in time. In this case, the photodiode acts as a converter of photons to photo-carriers as well as a capacitor storing the photo-carriers, until they are transferred and read by the Pixel’s Source Follower. Therefore, in this case, the accumulative voltage should be considered in the SNR calculations.

### 4.1. Numerical Modeling of the Results

The uniqueness of the suggested model is that it allows SNR analysis for any system with the same electrical network as shown in Figure 3, for any given illumination function ϕ(t). In Section 3, general expressions for SNR for both methods are calculated in Equations (11) and (13). The special case where the illumination function is a step function is also calculated in Equations (12) and (14).

**Figure 4:**
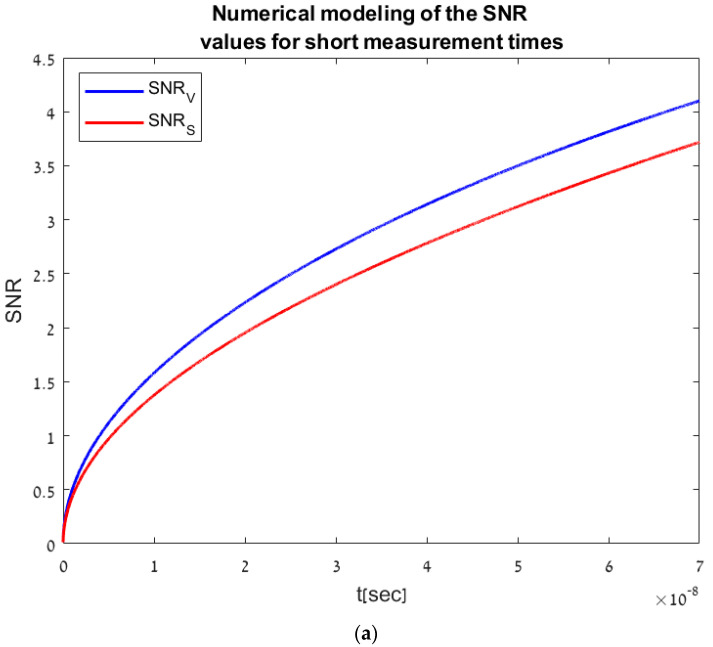

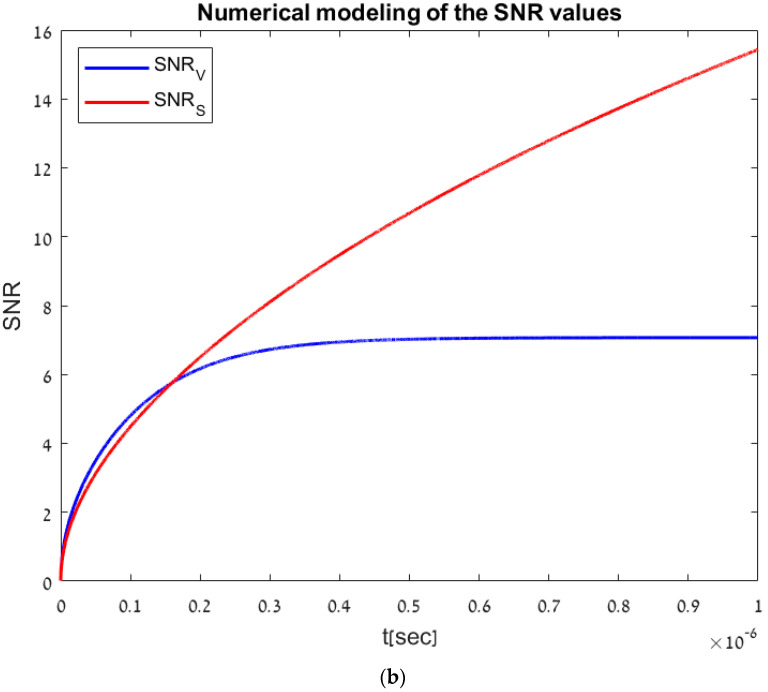
(**a**): The SNR values for measuring short times of up to 70 [ns], with square root trend of both methods, assuming $\phi_{0}={\left(4\;\left[ns\right]\right)}^{-1}$. (**b**): The SNR values for longer measuring times, with square root trend of the accumulative method and convergence of the temporal method, assuming $\phi_{0}={\left(4\;\left[ns\right]\right)}^{-1}$.

For typical values of $R=100\left[M\Omega \right]$ [14] and $C=1\left[fF\right]$ [11], the resulting α=1/RC value is $10^{7}\;\left[s^{-1}\right]$. For example, to measure depth in a range of up to 10 m, the pulse travelling time is $t=2\cdot 10\left[m\right]/3\cdot 10^{8}\left[\frac{m}{s}\right]=66[ns]$, resulting in αt<1. From Figure 4a, showing the SNR values for measuring times of up to 70 [ns], it is noticeable that the SNR values are higher in the temporal voltage method than the accumulative method, as expected due to the short-time pulse characterization of this method. For longer measurement times, meeting the condition αt≫1, as seen in Figure 4b, the temporal method SNR values converge into a constant value whereas in the accumulative method the SNR values keep its square root trend, resulting in a higher SNR value, meaning that the accumulative method is more suitable for longer measurement times—the higher the measuring time is, the more photo-carriers are created in the photodiode, resulting in a higher SNR.

### 4.2. Monte Carlo Simulations

**Figure 5:**
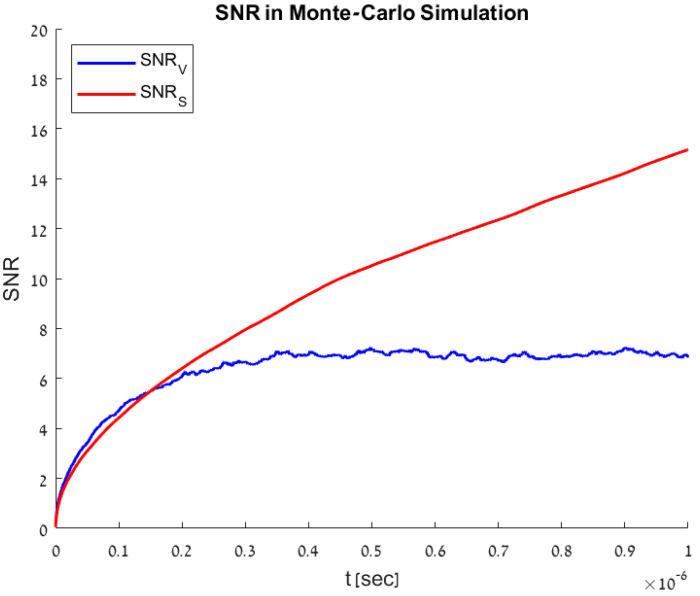
SNR values of the Monte Carlo simulation, based on 1000 repeated randomizations of the model described in Section 3.

To validate the model results, a Monte Carlo simulation [15] was performed on the model described in Section 3. The simulation was composed of 1000 realizations, each randomizing the total amount of photo-carriers injected into the model according to Poisson distribution and the time in which each of the photo-carriers were injected into the model. The model response was then calculated for both temporal and accumulation methods according to Equations (3)–(5). Mean and variance values over all the realizations were calculated, from which SNR values were extracted. The Monte Carlo simulation results are shown in Figure 5. These results match the numerical modeling detailed in Section 4.1 and shown in Figure 4b.

## 5. Summary

This study presents a new stochastic model for the case of gated imaging, where non-stationary conditions prevail. The model is based on fundamental physical assumptions. In an earlier paper we analyzed conversion gain of CMOS image sensors based on a stochastic approach. The conversion gain of an imager is best analyzed under stationary conditions. However, in the case of gated imaging, due to the non-stationary nature of the measuring conditions, the signal-to-noise ratio requires extending this approach. The new model is validated using a Monte Carlo simulation.

Improvements in efficiency and size shrinkage of laser sources, as well as technological advances in CMOS sensor technology, in particular CMOS SPAD, have brought the laser-gated imaging to be an established and mature technology. A laser pulse illuminates the scene, functioning as a source of light. The laser’s photons travel towards the object and then some of them are reflected towards the CMOS image sensor. The basic principle of gated imaging is to start the sensor’s exposure only when the reflected photons should return to the sensor, minimizing the sensing time of the photons as much as possible, preventing sensing of parasitic light sources and scattered photons. The gating time sets the depth of view of the scene.

Since in gated imaging the exposure is taking place after a delay from the pulse sending and for a short period of time, the stationary assumption of the earlier paper was revisited, and the SNR value was calculated under non-stationary conditions. The SNR can be measured in two different methods. The first method is based on temporal voltage, which is applied for very short illumination pulses. The second method is based on the accumulated, namely integrated voltage. The second method produces higher SNR values for longer measurement periods, while the first method produces higher SNR values for short measuring.

It should be noted that gating reduces only the background noise. The noise contributed by the sensor is integrated and in fact limits the useful gating time. This contribution is not considered in this study, in order to let the reader get used to the concepts of the modeling.

## Data Availability

Not applicable.
